# Intervention and Identifiability in Latent Variable Modelling

**DOI:** 10.1007/s11023-018-9460-y

**Published:** 2018-03-30

**Authors:** Jan-Willem Romeijn, Jon Williamson

**Affiliations:** 1 Faculty of Philosophy, University of Groningen, Oude Boteringestraat 52, 9712 GL Groningen, The Netherlands; 2 Department of Philosophy Cornwallis North West, University of Kent, Canterbury, Kent CT2 7NF UK

**Keywords:** Interventions, Statistical inference, Identifiability, Latent variable modelling

## Abstract

We consider the use of interventions for resolving a problem of unidentified statistical models. The leading examples are from latent variable modelling, an influential statistical tool in the social sciences. We first explain the problem of statistical identifiability and contrast it with the identifiability of causal models. We then draw a parallel between the latent variable models and Bayesian networks with hidden nodes. This allows us to clarify the use of interventions for dealing with unidentified statistical models. We end by discussing the philosophical and methodological import of our result.

## Introduction

A statistical model may include hypotheses that have identical likelihood functions over the entire sample space. This is the problem of statistical identifiability: several statistical hypotheses fit the data equally well, hence we cannot identify the best one by data alone. So-called unidentified models exhibit a form of underdetermination, though not the radical form that often features in arguments against scientific realism. The standard response to underdetermination is to look for theoretical criteria, such as simplicity or explanatory force, that help us choose between the rivals. In factor analytic models, for example, one might use criteria pertaining to the variation among the estimations of the statistical parameters to force a unique solution of the estimation of factor loadings.

In this paper we investigate a particular solution to the problem of statistical identifiability in the context of causal modelling. Given the context, let us stress that the statistical identifiability problem must not be confused with the problem of identifying so-called *causal effects* (cf. Pearl 2000, chapter 3). The latter concerns the determination of how a system responds to interventions, i.e., determining causal structure. Statistical identifiability is different because it does not involve uncertainty about causal structure. Instead it concerns the determination of statistical parameters within a model whose causal structure is fully specified. It occurs when the statistical hypotheses under consideration say the very same things about what observations to expect, i.e., they have exactly the same likelihood functions and thus perform equally well on the observed data.

That said, the solution that we investigate does rely on the causal interpretation of the statistical models. In fact, the solution assumes that certain aspects of the causal model are known, and therefore that the problem of causal identifiability has to some extent been resolved. It trades on the fact that the otherwise identical statistical hypotheses need not be equivalent in a causal sense. We can consider specific changes to the setup of the study, i.e., specific interventions, such that the hypotheses get different likelihood functions over the additional results. The hypotheses are then told apart by their differing causal content. For this solution to work, we need to presume that we have already determined how the system behaves after intervention.

Our solution to statistical identifiability conveys two messages. The first is philosophical: we want to bring to the fore an important and, to our mind, undervalued aspect of scientific confirmation, namely the use of intervention data. We believe that insights from the philosophy of experiment (e.g. Hacking 1980; Gooding 1990) can come to fruition in confirmation theory and we hope to make a modest start with that. A further message is methodological: we hope to contribute to a better understanding of the benefits of interventions and stimulate the uptake of statistical tools for modeling interventions in social science. Despite the availability of statistical theories and methodological tools for exploiting intervention data, scientists are often not aware of their potential. Moreover, insofar as there is awareness, this mostly concentrates on the identification of causal effects or the use of intervention data for determining causal structure (e.g., Spirtes et al. 2001; Eberhardt et al. 2010; Hyttinen et al. 2012; Silva and Scheines 2003). This paper suggests a different use of intervention data.

We present our argument in the setting of latent variable modelling, a statistical modelling tool from the social sciences that remains understudied in the philosophy of science, with one or two exceptions. Johnson (2014) offers a wonderful overview of the philosophical import of factor analysis in connection to the problem of underdetermination. Interestingly, although our papers target different problems and were written independently, they reach similar general conclusions. Factor analysis makes another appearance in Haig (2005) and Schurz (2008), namely as a model for abductive inference, and thus as a tool for generating and selecting theory. In this paper we take a different perspective. We employ exploratory factor analysis as an illustration of a more general problem concerning statistical unidentifiability, and we focus on the role of interventions in resolving it.

The paper is set up in the following way. In Sect. 2 we introduce statistical identifiability abstractly and in Sect. 3 we make these problems concrete for latent class analysis and factor analysis. We show in Sect. 4 that latent variable modelling is for our purposes identical to estimating parameters in a Bayesian network with hidden nodes. Just as is the case with causal Bayesian networks, data obtained after intervention can be used to identify features of models in factor analysis. In particular, we argue in Sect. 4.3 that intervention data can, under the right conditions, be used to resolve problems of statistical identifiability. In Sect. 5, finally, we briefly suggest how this model for intervention may prove useful to the philosophy of science in general.

We see the topic of this paper as an opportunity for a fruitful interaction between philosophers of science and social science methodologists. Our own expertise is first and foremost in the former: we mostly consider identifiability problems and causal models from an abstract point of view. Social science methodologists, on the other hand, regularly encounter such problems in practice. We believe that insights from the applications can shed valuable light on the theoretical problem. Similarly, we hope that our more theoretical insights will be of use to the methodologists.

## Unidentified Models

In what follows we characterize the problem of unidentified statistical models, and make it precise for latent class analysis (LCA), a well-known statistical technique in, e.g., psychometrics. LCA is a close cousin to factor analysis (FA). LCA and FA are both routinely used to interpret psychological test data, and working psychologists face the problem that the data often do not allow for a complete determination of the underlying classes or factors. This presents psychological science with its own version of the philosophical problem of underdetermination (cf. Johnson 2014).

### Identifiability in Statistics

Here we illustrate the concept of statistical identifiability using some toy examples. A more realistic setting will be introduced in Sect. 2.2.

Consider a simple statistical problem, in which we estimate the chances of events in independent and identical trials, e.g., results in psychological tests. An observation at time *t* is denoted by the assignment of a value to a binary variable $Q_{t}$, with possible values failing and passing the test. We denote a sequence of *t* observations or test results by means of the variable $S_{t}$. For example, if $S_{t} = 010\ldots 1$, then $Q_{1} = 0$, $Q_{2} = 1$, and so on. The hypothesis $H_{\theta }$ says that the chance of observing $Q_{t+1} = 1$ is $\theta$ irrespective of which sequence of outcomes $S_{t}$ precedes it.

1 $$\begin{aligned} P(Q_{t + 1} = 1 | H_{\theta }, S_{t}) = \theta \end{aligned}$$

for every $S_{t}$ and for each trial $Q_{t+1}$, an expression involving what is often called the *likelihood function* of $H_{\theta }$.^1^

The chance $\theta$ of the event $Q_{t + 1} = 1$ may be any value in [0, 1], so we have a whole continuum of hypotheses $H_{\theta }$ gathered in what we call a statistical model, denoted $\mathcal H$. On the basis of some sequence of events $S_{t}$, we can provide an estimate of $\theta$. We can do so either by defining a prior $P(H_{\theta })$ and then computing a posterior by Bayesian conditioning, or by defining an estimator function over the event space, typically the *observed relative frequency*:

$$\begin{aligned} {\hat{\theta }}(S_{t}) = \frac{1}{t} \sum _{i = 1}^{t} Q_{i}, \end{aligned}$$

The above estimation problem is completely unproblematic. The observations have a different bearing on each of the hypotheses in the model, i.e. each member of the set of hypotheses. If there is indeed a true hypothesis in the set, then according to well-known convergence theorems (cf. Earman 1992, pp. 141–149), the probability of assigning a probability 1 to this hypothesis will tend to one. In the limit, we can therefore almost always, in the technical sense of this expression, tell the statistical hypotheses apart.^2^

This situation is different if we take a slightly different set of statistical hypotheses $G_{\xi }$, characterized as follows:

$$\begin{aligned} P(Q_{t + 1} = 1 | G_{\xi }, S_{t}) = \xi ^{2}, \qquad \xi \in [-\,1, 1]. \end{aligned}$$

This set of hypotheses covers the same set of possibilities, only they are doubly labelled. The hypotheses $G_{\xi }$ and $G_{-\xi }$ are indistinguishable, because they both assign exactly the same probability to all the observations: $P(Q_{t + 1} = 1 | G_{\xi } \cap S_{t}) = P(Q_{t + 1} = 1 | G_{-\xi } \cap S_{t})$. In such a case, we speak of an *unidentifiable* model. Notice that this situation is much like having a single equation with two unknowns, for instance $x + y = 1$ with $x, y \in [0, 1]$. We cannot find a unique solution for *x* and *y*, rather we have a whole collection of solutions. To force uniqueness, we need a further equation, e.g., $x - y = 0$.

Unidentifiable models are in a sense underdetermined by the observations. Importantly, this kind of statistical underdetermination is not of the kind most feared by scientific realists, because there may well be experiments or additional observations that would allow one to disentangle the statistical hypotheses. This paper shows how additional experiments can achieve this.

### Latent Variable Models

The above example of statistical underdetermination is rather contrived: no reason is given for distinguishing between the regions $\xi > 0$ and $\xi < 0$. However, there are cases in which it makes perfect sense to introduce distinctions between hypotheses that do not differ in their likelihood functions. This subsection is devoted to presenting one of these cases, involving a so-called latent class model. The exposition is partly borrowed from [omitted for purpose of blind review].

A latent variable model posits hidden, or latent, random variables on the basis of an analysis of the correlational structure of observed, or manifest, random variables. Examples are latent class models, which are discussed below, and factor models, in which latent and manifest variables are continuous.^3^ Suppose that in some experiment we observe (continuously or discretely varying) levels of fear *F* and loathing *L* in a number of individuals who are represented via the index *i*, and we find a positive correlation between these two variables,

$$\begin{aligned} P(F_{i}, L_{i}) > P(F_{i}) P(L_{i}). \end{aligned}$$

One way of accounting for the correlation is by positing a statistical model over the variables in which fear and loathing may be related directly.

We may feel that it is neither the loathing that instills fear in people, nor the fear that invites loathing. Instead we might think that both feelings are correlated because of a latent characteristic of the individuals, namely a depression from they might be suffering. Conditional on the level of the depression, denoted $D_{i}$, fear and loathing might be uncorrelated:

$$\begin{aligned} P(D_{i}, F_{i}, L_{i}) = P(D_{i}) P(F_{i} | D_{i}) P(L_{i} | D_{i}). \end{aligned}$$

In the case in which all the variables vary continuously, we speak of a *factor model*. We then say that the depression is the *common factor* to the observable, or manifest, variables of fear and loathing, and the correlations between the depression variable and the levels of fear and loathing we call the *factor loadings*.

Latent variable models come in several shapes and sizes, subdivided according to whether the manifest and latent variables are categorical or continuous. In what follows we discuss one of the most straightforward applications of such models, in which both the manifest and latent variables are binary: latent class analysis. Our reason is that we are making a conceptual point about interventions and underdetermination. For this purpose the simplest format of factor analysis suffices.

To illustrate the latent class analysis, say that the depression is either present in subject *i*, $D_{i} = 1$, or absent, $D_{i} = 0$, and similarly for fear and loathing. We assume that over time the variables are independent and identically distributed. That is, for $i \ne i'$ the variable $D_{i}$ is independent of $D_{i'}$, $F_{i'}$ and $L_{i'}$, and similarly for $F_{i}$ and $L_{i}$. Out of the possible probabilistic dependencies among $F_{i}$, $L_{i}$ and $D_{i}$, we confine ourselves to

2 $$\begin{aligned} P(F_{i} = 1 | D_{i} = j)&=  \phi _{j}, \end{aligned}$$

3 $$\begin{aligned} P(L_{i} = 1 | D_{i} = j)&=  \lambda _{j}, \end{aligned}$$

for $j = 0, 1$, a conditional version of the Bernoulli model of Eq. (1). Similarly for the variable $D_{i}$,

4 $$\begin{aligned} P(D_{i} = 1) = \delta \end{aligned}$$

The probability over the variables $D_{i}$, $L_{i}$ and $F_{i}$ is thus given by five Bernoulli distributions, each characterized independently by a single chance parameter.

There may be experimental conditions in which the latent class that enhances or reduces fear and loathing is observable, e.g., when the individuals all take a drug *E* which reduces fear and loathing. But the depression variable *D* in our example is latent: it cannot be observed directly. Although the causal or mechanistic underpinning is unknown, we might nevertheless posit such a variable. Exploratory factor analysis is a technique for arriving at such common factors in a systematic way, in cases where the variables aer continuous. When given a set of correlations among manifest variables, it produces a statistical model of latent common factors that accounts for exactly these correlations.^4^

Perhaps unsurprisingly, latent variable models suffer from problems of identifiability. They posit the theoretical structure of unobservable common causes, over and above the observed correlations between observable variables. There will generally be many latent variable models, and accordingly many different causal structures, that fit the data. This is the problem of causal identifiability alluded to earlier. However, even if all modeling choices have been made and if the list of salient variables and their causal structure have been fixed, either by assumption or by background knowledge, the problem of statistical underdetermination may appear. In what follows we focus specifically on this restricted identification problem.

### Unidentifiability of Latent Variable Models

We now show that the model of Eqs. (2), (3) and (4) cannot be identified by the data.

Focus on the dimensions of this model. We count 5 parameters, namely $\delta$, and $\phi _{j}$ and $\lambda _{j}$ for $j = 0, 1$. On the other hand, we have the binary observations $F_{i}$ and $L_{i}$ that can be used to determine these parameters. But because we are using Bernoulli hypotheses, only the observed relative frequencies of the possible combinations of $F_{i}$ and $L_{i}$ matter. And because we have 4 possible combinations of $F_{i}$ and $L_{i}$, whose relative frequencies must add up to 1, we have 3 frequencies to determine the 5 parameters in the model. After having used the observations in the determination of the parameters, therefore, we still have 2 degrees of freedom left. Hence the values of the parameters in the model cannot be determined by the observation data uniquely.

We can state this problem in more detail by looking at the likelihoods for the observations of possible combinations of $F_{i}$ and $L_{i}$. We write $\theta = \langle \delta , \phi _{0}, \phi _{1}, \lambda _{0}, \lambda _{1} \rangle$. For the likelihoods we write

5 $$\begin{aligned} P(F_{i} = 0, L_{i} = 1 | H_{\theta }) &=  \theta _{01} = \delta (1 - \phi _{1}) \lambda _{1} + (1 - \delta ) (1 - \phi _{0}) \lambda _{0}, \nonumber \\ P(F_{i} = 1, L_{i} = 0 | H_{\theta }) &=   \theta _{10} = \delta \phi _{1} (1 - \lambda _{1}) + (1 - \delta ) \phi _{0} (1 - \lambda _{0}), \nonumber \\ P(F_{i} = 1, L_{i} = 1 | H_{\theta }) &=  \theta _{11} = \delta \phi _{1} \lambda _{1} + (1 - \delta ) \phi _{0} \lambda _{0}, \end{aligned}$$

where we omitted mention of the other individuals $S_{i-1}$. The fourth likelihood, $P(F_{i} = 0, L_{i} = 0 | H_{\theta })$, can be derived from these expressions. The salient point is that the system of equations resulting from filling in particular values for the above likelihoods has infinitely many solutions in terms of the components of $\theta$: for any value of the likelihoods, the space of solutions in $\theta$ has 2 dimensions. The statistical model is thus unidentifiable.

Let us briefly elaborate on the unidentifiability of the model. It means that the likelihood function over the model does not have a unique maximum, and so that the maximum-likelihood estimator does not point to a uniquely best hypothesis. In fact there are infinitely many hypotheses compatible with the data. Say that we observe the following relative frequencies:

6 $$\begin{aligned} r_{11} = \frac{1}{t} \sum _{i = 1}^{t} F_{i} L_{i}, \qquad r_{10} = \frac{1}{t} \sum _{i = 1}^{t} F_{i} (1 - L_{i}), \qquad r_{01} = \frac{1}{t} \sum _{i = 1}^{t} (1 - F_{i}) L_{i}. \end{aligned}$$

The likelihood $P(S_{t} | H_{\theta })$ is maximal if the observed relative frequencies $r_{jk}$ match the corresponding likelihoods $\theta _{jk}$ for all *j* and *k*:

7 $$\begin{aligned} \theta _{jk} = r_{jk}. \end{aligned}$$

But as said, there are infinitely many hypotheses $H_{\theta }$ that have these particular values for the likelihoods. Consequently, there is no unique hypothesis $H_{\theta }$ that has maximal overall likelihood $P(S_{t} | H_{\theta })$.

For future reference we note that, by means of the likelihoods given in Eqs. (5), we can determine a posterior probability for the hypotheses in the model, $P(H_{\theta } | S_{t})$. And from the posterior distribution over the hypotheses we can generate the expectation value of the parameter $\theta$ of the model $\mathcal H$, according to

8 $$\begin{aligned} \text {E}[\theta ] = \int _{\mathcal{H}} \theta \, P(H_{\theta } | S_{t})\, d\theta . \end{aligned}$$

Here $\theta$ runs over $[0, 1]^{5}$ because the model is spanned by five independent chances. Like the posterior, the estimations will suffer from the fact that the hypotheses cannot be told apart: they will depend on the prior probability over the hypotheses. Of course, this is usually the case in a Bayesian analysis. What is troublesome is that no amount of additional data can eliminate this dependence of the estimations on the prior.

One reaction is to downplay the identifiability problem and say that it only concerns the values of these abstract parameters and not the empirical consequences. But because the estimations and expectations are not fully determined, the nature of the latent variable underlying the manifest variables is not determined either: it is not clear what causal role it plays. Different values for the parameters $\phi _{j}$ and $\lambda _{j}$ entail different systematic relations between depression, fear and loathing, and ultimately this reflects back on our understanding of the posited notion of depression itself.

## Identifiability in Multivariate Linear Regression

The foregoing mostly concerned a latent class model, and such models are a lot simpler than the models of factor analysis. In this section we argue that the problem outlined above also shows up there. Furthermore, we will note that in factor analysis there are actually two statistical identifiability problems. The first is made more concrete in the first subsection. It presents an analogous problem to that described in Sect. 2.3. The second type is briefly mentioned in the second subsection, mostly because it has been hotly debated in psychological methodology, but also because the present paper can offer a specific angle on it.

### The Rotation Problem

In factor analysis the variables are not binary but continuous, the probabilistic relations between the variables are linear regressions with normal errors, and the latent variable is assumed to be governed by some continuous distribution as well. In our example we may write $F_{i} = f$ for the event that the level of fear is $f \in \mathbb {R}$, and similarly for depression $D_{i} = d$. Then the relation between $F_{i}$ and $D_{i}$, for example, is

9 $$\begin{aligned} P(F_{i} = f | D_{i} = d) = N(\lambda _{F} d, \sigma _{F}) \end{aligned}$$

in which $N(\lambda x, \sigma )$ is a normal distribution over the values *f* of $F_{i}$. So the relation between the variables $D_{i}$ and $F_{i}$ is characterized by a richer family of distributions, parameterized by a regression parameter $\lambda _{F}$ and an error of size $\sigma _{F}$.

Despite these differences, the same kind of statistical identifiability problems occur. Note that we can extend factor models like the one above to include any number of common factors. However, once a model includes more than one common factor, we find that the factor loadings are not completely determined. Suppose, for example, that we analyze fear *F*, loathing *L*, and sleeplessness *S* in terms of two common factors, one of them depression *D*, and the other the latent variable *C*. Every individual is supposed to occupy a specific position in the $C \times D$ surface. We might feel that a more natural way of understanding the surface of latent variables is by labeling the states in this surface differently, for example by introducing variables *A* and *B*, both of which are linear combinations of *C* and *D*. The factors in a model may be linearly combined or, in more spatial terms, rotated to form any new pair of factors.^5^

The problem with this is that any rotation of factors, e.g., from $\{ C, D \}$ to some $\{ A, B \}$, will perform equally well on the estimation criterion, be it maximum likelihood, generalized least squares, or similar, as long as we can adapt the factor loadings and perhaps the correlations among the factors accordingly. This problem is known as the problem of the rotation of factor scores. Neither the estimation criteria—often maximum likelihood—nor Bayesian methods of incorporating the data lead to a single best hypothesis in the factor model. The result is rather a collection of such hypotheses that all fit optimally. That is, the factor model is not identifiable.

A standard reaction to the rotation problem is to adopt further theoretical criteria that can constrain the latent variables. For example, it may be considered desirable to have maximal variation among the regression coefficients which, intuitively, comes down to coupling each latent variable with a distinct subset of manifest variables.^6^ The thing to note is that, from the point of view of statistics, the choice for how to parameterize the space of latent variables is underdetermined: we cannot decide between these parameterizations on the basis of the observations alone.

In this paper we will not elaborate the mathematical details of identifiability problems in these more complicated models. For present purposes, it suffices to use the simpler factor model of Eqs. (2) to (4). The crucial characteristic in all of what follows is that there are latent variables explaining the correlational structure among the manifest variables, and that these structures are not fully determined by the correlations among the manifest variables. Admittedly, this paper thereby falls short of providing practical guidelines for dealing with the rotation problem, but we hope that our suggestions about a means to remedy it are valuable in their own right.

### Factor Score Indeterminacy

There is another problem with factor analysis that can be framed as an identifiability problem, and that has received considerable attention within statistical psychology.^7^

Say that we have rotated the factors to meet the theoretical criterion of our choice. Can we then reconstruct the latent variable itself, that is, can we provide a labeling in which each individual, i.e., each assignment of values to the observable variables, is assigned a determinate expected latent score? Sadly, the classical statistical answer here is negative. We still have to deal with the so-called *indeterminacy of factor scores*, meaning that there is a variety of ways in which we can organize the allocation of the individuals on the latent scores, all of them perfectly consistent with the estimations.^8^

The type of unidentifiability presented by factor score indeterminacy depends on what we take to be the statistical inference underlying factor analysis. In the context of this paper, we take the factor analysis to specify a complete probability assignment over the latent and manifest variables, including a prior probability over the latent variables. As explained in Bartholomew and Knott (1999), factor score indeterminacy is thereby eliminated, as long as there are sufficiently many manifest variables that are related to the latent variables according to distributions of a suitable, namely exponential, form. In this paper we will therefore ignore most of the discussion on factor score indeterminacy.

There is one point at which the problem of factor score indeterminacy enters the present discussion. We will show in what follows that intervention data can also be used to choose among a class of priors. But as indicated, the problem of choosing a prior probability is related to the problem of factor score indeterminacy. Therefore the use of intervention data, which resolves the identifiability problem discussed above, provides a new perspective on the problem of the indeterminacy of factor scores as well. We will return to this idea in Sect. 5.2.

## Interventions to Resolve Identifiability

In the foregoing we have shown that latent variable models suffer from identifiability problems. We now explain these problems by revealing analogous problems in the estimation of parameters in Bayesian networks. This leads us to consider a specific solution, namely by means of intervention data. We first introduce Bayesian networks in Sect. 4.1, then the notion of intervention in Sect. 4.2, and finally its use in identifying latent variable models in Sect. 4.3. To the best of our knowledge, this solution to the problem of statistical identifiability has not yet been offered in the literature. The fact that the solution is not worked out in full generality here is hopefully compensated for by the fact that it offers a new insight into the use of intervention data.

### Bayesian Networks and Factor Analysis

In general, a Bayesian network consists of a directed acyclic graph on a finite set of variables $\{ D, F, L, E \ldots \}$ together with the probability distributions of each variable conditional on its parents in the graph, e.g., $P(E \mid Par _E)$. The graph is related to the probability distribution over the variables by an assumption known as the Markov Condition: each variable is probabilistically independent of its non-descendants in the graph, conditional on its parents, e.g., ; see Pearl (2000). Under this assumption the network suffices to determine the joint probability distribution over the variables, via the identity:

10 $$\begin{aligned} P(D, F, L, \ldots ) = P( D \mid Par _D) \times P( F \mid Par _F) \times P(L \mid Par _L) \times \ldots \end{aligned}$$

The probability of any assignment of values to the variables on the left hand side of this equation can be computed by filling in these valuations on the right hand side.

It is well-known that Bayesian networks and latent variable modeling are closely related. In fact, the introduction of the latent class models for the binary variables $\{ F, L, D \}$ was already an introduction to a specific class of Bayesian networks. To ease exposition, suppose that there are no inter-subject dependencies and that the same probability assignment describes all subjects,

11 $$\begin{aligned} P(D_{i}, F_{i}, L_{i}) = P(D_{i'}, F_{i'}, L_{i'}), \end{aligned}$$

so that we can omit the subscripts *i*. For each subject, the factor analysis determines a probability function *P*(*F*, *L*, *D*) that satisfies a specific symmetry: conditional on the latent depression *D* there is no correlation between the manifest fear *F* and loathing *L*,

12 $$\begin{aligned} P(D, F, L) = P(D) P(F | D) P(L | D). \end{aligned}$$

**Fig. 1:**
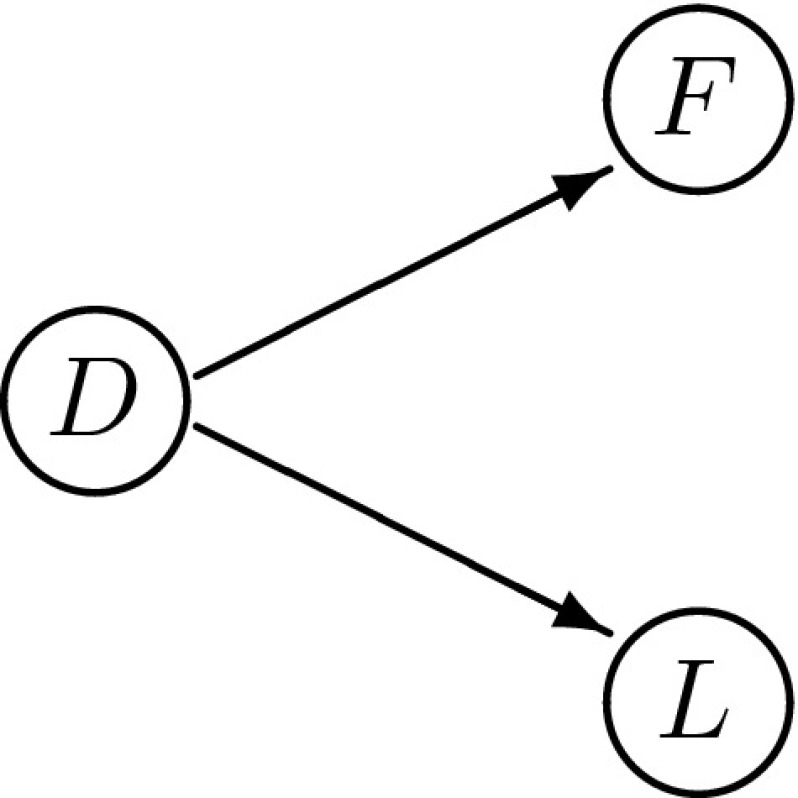
The graphical structure representing the independence relations in a factor analysis of depression, fear and loathing

On this basis we can build a network, with the variables *F*, *L* and *D* as nodes. The probability function determined by factor analysis can thus be represented in a Bayesian network whose graph is depicted in Fig. 1.

There are also differences between the theory of Bayesian networks and that of latent variable models. For one, the latter entails a rather specific network structure: there are hidden parent nodes, observable child nodes, there are typically fewer parents than children, and any child can be connected to any parent. On the other hand, applications of the former are usually restricted to probability functions over finite or at least countable domains. Nodes with continuous domains are not that commonly discussed, although they have been studied in the context of structural equation models, for example by Pearl (2000), and, from the side of latent variable modeling, by von Eye and Clogg (1994). A related difference is that in most applications of factor analysis, the probability functions that are considered are restricted to normal distributions over latent nodes, and to linear regressions with normal errors between latent and observable nodes. Applications of Bayesian networks are typically, but not necessarily, restricted to Bernoulli distributions.

In this section we approach latent variable modelling from the angle of Bayesian networks, using the framework for inference over Bayesian networks presented in Romeijn et al. (2009). Hence the statistical underdetermination presented in Sect. 2.3 is framed as a problem to do with determining the posterior probability distribution over the parameters that characterize the Bayesian network of Fig. 1. The aim is to resolve this statistical underdetermination by means of intervention data. In order to do this, we first introduce interventions in the context of Bayesian networks.

### Interventions

A causally interpreted Bayesian network, or causal net for short, is a Bayesian network where the graph is interpreted as a causal graph. That is, each arrow in the graph is interpreted as denoting a direct causal relationship from the parent variable to the child variable. Under this interpretation, the Markov Condition is called the Causal Markov Condition. It says that each variable is probabilistically independent of its non-effects conditional on its direct causes. It is often assumed that the Causal Markov Condition is bound to hold if the graph in the net is correct and is closed under common causes (i.e., any common causes of variables in the net are also included in the net). While there are situations in which the Causal Markov Condition is implausible, it can nevertheless be justified as a default assumption (Williamson 2005), and we shall take it for granted here.

Causal nets are helpful for predicting the effects of interventions. When an experimenter intervenes to fix the value of a target variable, she interrupts the normal course of affairs and sets the variable exogenously. The usual mechanisms by which the target variable is determined are thereby replaced by new mechanisms; these new mechanisms allow the experimenter to fix a value of the variable. An ‘ideal’ or ‘divine’ intervention is one in which the intervention only changes the intended target variable, without changing other variables under consideration and without changing other causal relationships under consideration. By means of Eq. (10) we can determine the probability $P'$ that some variable *F* takes value 0 after an ideal intervention has been performed that sets *D* to 1, say. Note that the causal net determines two different probability distributions, *P* before and $P'$ after intervention. While *P* and $P'$ will coincide in the probability assignments to non-descendants of *D*, and also in the probability assignments conditional on *D*, the unconditional probabilities for the variables downstream from *D* will be different.

Not all interventions are ideal. Other forms of intervention are *ham-fisted* in that they change the values of several variables at once, or *non-modular* in that they change other causal relationships, or *parametric* in the sense that they change the conditional probability distribution of the target variable without deterministically fixing its value. One subspecies of parametric intervention, which we shall refer to as a *stochastic intervention*, is central to our concerns: an intervention in which one sets the probability of the target variable to a new value $P'(D = 1) = \delta '$ while leaving the rest of the network intact. In other words, the causal net is transformed by eliminating arrows into the target *D*, setting its unconditional distribution to $P'(D = 1) = \delta '$, and then determining the new probabilities for other variables.^9^

**Fig. 2:**
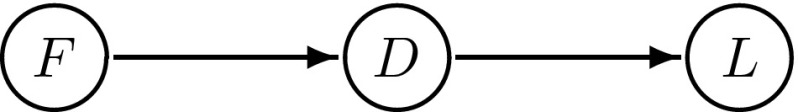
A chain of fear *F* causing depression *D*, which causes loathing *L*

**Fig. 3:**
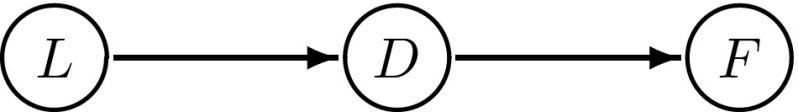
A chain of loathing *L* causing depression *D*, which causes fear *F*

Generally, interventions can help with identifiability problems in two ways. First, they can help with the identifiability of causal effects, as alluded to at the start of this paper. If more than one causal structure is compatible with evidence or if the specific relation between two variables is not known, then one can intervene, collect more evidence, and use this new evidence to decide over the matter. To take the example presented in the foregoing, suppose variables *F*, *L* and *D* are all measured, and that the resulting data shows that *F* and *L* are probabilistically independent conditional on *D*, written . This evidence is compatible with the causal graph of Fig. 1, but equally with Figs. 2 and 3. The evidence can be used to fill in the conditional probability distributions on these causal models, but cannot decide between them. An intervention can decide between them, however. If, after intervening to change the distribution of *D*, the distribution of *F* and *L* are changed, then that favours Fig. 1. Otherwise if only the distribution of *L* is changed after intervention, then Fig. 2 is supported, and if only the distribution of *F* is changed then Fig. 3 is supported.

By contrast, the point of this paper is that interventions can be used to make a statistical model with a given causal structure identifiable. Suppose that the causal structure is known and that data is collected which helps to estimate the probability distributions of some variables conditional on their parents, but which does not determine conditional distributions that attach to other variables. By carrying out an intervention, an experimenter changes the conditional distribution of one variable without changing the distributions of other variables. The data obtained after the intervention can then be used in conjunction with the old data to further constrain the values of the underdetermined distributions.

### Interventions and Model Identifiability

In this section we show how interventions, in a wide reading of this term, can be used to resolve the statistical identifiability problem for latent class models, introduced in Sect. 2.3 with the example on depression, fear, and loathing.

Let us briefly explain the general idea. We need to assume that the latent variable model is more than a convenient way of representing the probability functions involved. The arrows in the model need to be interpreted causally, that is, the latent variables must be taken as the causes of the observed variables. With this causal assumption in place, an intervention on the subjects will indeed change the distribution over the latent variables of the subjects. Importantly, in the application of interventions that we are currently considering it is not required that we have detailed knowledge of how the intervention has influenced the target variable, as long as we know that this change is not an effect of other variables in the model.

Note, in particular, that a stochastic intervention is taken to be modular: the probabilistic relations between the latent and the manifest variables does not change as a result of the intervention. As explained in the foregoing, after an intervention we obtain an entirely new estimation problem for the parameters in the Bayesian network. However, we assume that the parameters associated with the relations between latent and manifest variables do not change: the values of $\phi _{i}$ and $\lambda _{i}$ are not affected. In the following we show that, depending on the model, the data obtained after an intervention of this type can be used to select a unique best estimate for the parameter values in the latent variable model.

Consider again the model characterized by Eqs. (2) to (4), (11) and (12). As explained in the foregoing, an intervention is an exogenous change to the probability assignment. In this particular case, some change is made to the node *D*, e.g., all the subjects are given a treatment intended to change the probability for depression. We thus change the probability of depression, $P(D_{i} = 1) = \delta$, to a new value,

$$\begin{aligned} P'(D_{i} = 1) = \delta ', \end{aligned}$$

which—we assume—is less than than $\delta$. The relation of the depression variable to the variables of fear and loathing, given by $P'(F_{i} = 1 | D_{i} = j) = \phi _{j}$ and $P'(L_{i} = 1 | D_{i} = j) = \lambda _{j}$, is not changed by the intervention: the treatment changes the probability for depression but not how depression, whether absent or present, affects feelings of fear and loathing.

It is important to stress that the intervention under consideration covers a wider class than what is usually taken as an intervention in the literature of Bayesian networks. We do not need to suppose that the details of the exogenous change to the probability of depression is known but merely that it has particular qualitative characteristics, e.g., that $\delta ' < \delta$. Moreover, we need not even suppose that we only target the depression variable *D*. Any ham-fisted intervention that makes an exogenous change to other variables that are not causes of the observables under consideration, in addition to the change on the latent variable under consideration, is suitable as an intervention. This means that the solution of the statistical identifiability problem considered here may also work in the context of a so-called ‘natural experiment’.

After the intervention, or exogenous change to the system, we record the observations $S'_{t}$ in the same set of *t* individuals. By analogy to Eq. (6), we observe the numbers of the occurrences in the new sequence of observations $S'_{t}$,

$$\begin{aligned} r'_{11} = \frac{1}{t} \sum _{i = 1}^{t} F_{i} L_{i}, \quad \ldots. \end{aligned}$$

So $r'_{jk}$ are the relative frequencies of the variables *F* and *L* as observed after the intervention. They present three further constraints on the parameters of the latent variable model.

To get the point across quickly, we focus again on the dimensions of the model. This time we count a number of 6 parameters, namely $\delta$, $\phi _{j}$ and $\lambda _{j}$ for $j = 0, 1$, and finally $\delta '$. On the other hand, we have a richer set of observations that can be used to determine these parameters. Specifically, we have 3 observed relative frequencies of $f^{j}_{i} \wedge l^{k}_{i}$ before intervention, and 3 of them after intervention, so 6 in total. Whereas previously we had two degrees of freedom left after the incorporation of the data, we can now fill in all the parameter values of the factor model.

Let us make this more precise. As before, we have the likelihoods of Eqs. (5). But to these expressions we now add the likelihoods of the hypotheses after the intervention:

13 $$\begin{aligned} P'(F_{i} = 0, L_{i} = 1 | H_{\theta }) &=  \theta '_{01} = \delta ' (1 - \phi _{1}) \lambda _{1} + (1 - \delta ') (1 - \phi _{0}) \lambda _{0}, \nonumber \\ P'(F_{i} = 1, L_{i} = 0 | H_{\theta }) &=   \theta '_{10} = \delta ' \phi _{1} (1 - \lambda _{1}) + (1 - \delta ') \phi _{0} (1 - \lambda _{0}), \nonumber \\ P'(F_{i} = 1, L_{i} = 1 | H_{\theta }) &=  \theta '_{11} = \delta ' \phi _{1} \lambda _{1} + (1 - \delta ') \phi _{0} \lambda _{0}. \end{aligned}$$

The system of equations that results from equating likelihoods and observed relative frequencies before and after intervention is

14 $$\begin{aligned} \theta _{jk} = r_{jk} \qquad \text {and} \qquad \theta '_{jk} = r'_{jk} \end{aligned}$$

for all *j* and *k*. Each of these constrains the parameters in $\theta$ and $\theta '$ in a particular way.

The “Appendix” to this paper shows that if this system of equations has a solution, then the solution is unique up to a transformation of the two values for *D*. Solutions thus come in mirror-image pairs, differing in the interpretation of the values for the variable *D* or, in other words, differing in whether the intervention has beneficial or adverse effects on the probability of being depressed. On the assumption that the treatment reduces the probability for depression, every hypothesis $H_{\theta }$ in the model is associated with a unique set of values for the likelihoods $\theta _{jk}$ and $\theta '_{jk}$. The conclusion is that if the data are generated by a chance process specified by a hypothesis $H_{\theta }$, then we can identify this hypothesis, in the same way as we were able to identify the true $H_{\theta }$ in the model of Eq. (1).

Note that this does not hold for the entire range of possible values for the observed frequencies. For extremal values there is still an infinity of solutions. Moreover, certain combinations of frequencies simply do not match with any of the statistical hypotheses within the model. In those cases the intervention data overdetermine the latent variable model, i.e., it fails to fit all the correlations. We must then look for a richer statistical model. It would be rather natural to incorporate this aspect of scientific reasoning into our account, by describing how statistical models are adapted when intervention data yield a bad fit. The idea is that the overdetermination due to intervention may lead to controlled and formally specified changes in the model, and that this may lead to a formal account of theory change. However, such an account is beyond the scope of the current paper.

The main conclusion for now is that intervention data can indeed be used to resolve the identifiability problem introduced in Sect. 2.1. If there are parameter values matching the relative frequencies exactly, then on the assumption that the treatment is beneficial, these values are unique: the likelihood function has a unique maximum after the normal and the intervention data are incorporated. While we have only shown this for a simple example, it is readily seen, and briefly considered in the “Appendix”, that the example generalizes. The example serves as a proof of principle and supports the central idea of this paper, which is that interventions can help to resolve statistical identifiability problems.

## Philosophical and Practical Implications

We now discuss the philosophical and practical implications of the approach of this paper. After that we briefly revisit the indeterminacy of factor scores and suggest how intervention data can be used to resolve this indeterminacy, at least in the form it takes within a Bayesian statistical model.

### Interventions Replace Theoretical Criteria

Our paper suggests a novel way to use intervention data, namely to resolve statistical identifiability problems. Where we had otherwise to use a theoretical criterion to choose among the equally well fitting alternative hypotheses, we can now make this choice on the basis of additional data, obtained after intervention. One might say that within statistics the identifiability problem has fuzzy edges: it can be resolved by an appeal to theoretical criteria, as routinely done for the rotation problem in factor analysis, but it can also be resolved by extending the realm of observations to include intervention data.

It is worth reiterating that we do not need to know anything about the exact impact of the intervention. That is, we do not need to know the exact value of $\delta '$. It suffices that we have changed the probability of the latent variable. Clearly, this is not to say that the use of intervention data requires no assumptions whatsoever. As indicated in the foregoing, the new data can only be taken as pertaining to the same parameters if we assume that the causal structure of the latent and observed variables is, at least roughly, correct. More specifically, we need to assume that the probabilistic relations between the latent and the observed variables, expressed in $\phi _{i}$ and $\lambda _{i}$, remain invariant under intervention. So in order to employ the intervention data for a resolution of the identifiability problem, we have to make particular causal assumptions. In a sense these modeling assumptions help us to get more out of the data than would otherwise be possible.^10^

We think that this resolution by causal assumptions and further empirical data is preferable to a resolution that employs a theoretical criterion only. This may be interesting for philosophers concerned with the interplay between theory and empirical fact in confirmation relations. Additionally, the result may help to put latent variable modelling on a firmer footing—in particular factor analysis, which has long been regarded by some as somewhat speculative (Furfey and Daly 1937). Finally, the use of interventions to resolve identifiability problems in factor analysis may be of practical interest. The rotation problem is a live one for designers of clinical and personality tests: how do we relate clusters of test items to specific personality traits? And what traits should we distinguish in the first place? Our suggestion would be that intervention data may help constrain the latent structure behind psychometric tests, thereby providing a clearer view of what the tests are measuring.^11^

### Interventions and the Indeterminacy of Factor Scores

We briefly remark on the problem of the indeterminacy of factor scores, as discussed in Sect. 3.2. Insofar as there is a problem with factor scores in the Bayesian treatment, intervention data can play an interesting role.

Recall that the expected value $\text {E}[\theta ]$, given in Eq. (7), depends on the posterior probability over the parameter $P(H_{\theta } | S_{t})$, and that this posterior depends on the prior probability $P(H_{\theta })$. As shown by Bartholomew and Knott (1999), the indeterminacy of factor scores in classical factor analysis derives directly from the fact that a prior probability is not provided. And because in a Bayesian treatment such a prior is assumed, we can say that Bayesian factor analysis is not affected by factor score indeterminacy. However, the prior is assumed, not derived, so a classical statistician may well ask for a motivation of the prior probability assignment.

Following the ideas set out above, the prior probability may be determined by means of intervention data. Instead of choosing a single prior, we might consider a range of priors over the parameter values, labeled by $\rho$ say. We thereby increase the dimension of the parameter space by one. But we might know from a different study that the chance of being depressed after the treatment $\delta '$ has some particular value, or is functionally related to the chance on depression before treatment. This reduces the number of parameters by one again, because $\delta '$ is then fixed, or every $\delta '$ is coupled to a unique value $\delta$. The net effect is that we can again estimate all the parameters, namely $\delta$, $\phi _{j}$ and $\lambda _{j}$ for $j = 0, 1$, and finally the second-order parameter $\rho$.^12^

In other words, just as we can estimate the effects of an intervention, $\delta '$, we can estimate the prior probability assignment that best suits the factor model. Of course, this is just a simple example. We have not said anything about the more realistic continuous case, in which we typically assume a normal distribution over the continuous variable $D_{i}$ as prior. Moreover, it is unrealistic to suppose that there is a clear and deterministic relation between the parameters governing the distribution over the variables $D_{i}$ before and after the intervention. Nevertheless, we suggest that the analysis presented here illuminates how intervention data can be of use in dealing with the rightful heir of the problem of factor score indeterminacy in Bayesian factor analysis, namely the problem of how to choose a prior.

## Conclusion

In this paper we have investigated the use of interventions for the problem of statistical identifiability: if two hypotheses have exactly the same likelihoods for all the possible observations, then how do we choose between them? While an answer to this question often invokes theoretical criteria such as simplicity and explanatory considerations, we have provided a partial answer in terms of empirical criteria. The idea is to use the background theory that generates the hypotheses, namely the causal structure. This theory provides us with a recipe for how to deal with interventions. Together with some assumptions on the causal structure of the latent and observed variables, the intervention data enable us to tell the statistically equivalent hypotheses apart.

We illustrated the identifiability problem by means of a latent class model. That is, we showed how interventions can be framed in terms of alterations to such a model, and how the intervention data can then be employed. In this paper we have not developed the same ideas for the more practical setting of factor analysis with normal distributions over continuous variables. But we believe that the problems identified in discrete Bayesian networks is in all the relevant respects similar to the rotation problem in the continuous setting, and we suggest that future work can resolve this problem of rotation by appealing to intervention data. On the other hand we realize that there is still a long way to go from the theoretical considerations in this work to the practical concerns of psychometricians.^13^

We will mention one specific theme for future research. We suggested that, relative to a given causal structure that links latent and observable variables, intervention data can also guide extensions of the statistical model. The rough idea is that the specifics of the misfit between model and intervention data will suggest how the latent structure might be adapted to repair the fit. Model selection techniques and further considerations of complexity or conservativity might then determine which of these adaptations is most appropriate. The methods and algorithms for putting this idea to work have yet to be determined, but we think that there are many potential applications of the idea. A tool for guiding extensions of statistical models can be of use to experimental scientists, but also to computer scientists working on the automated search of network structures.

Such applications lie within the realm of statistical methodology. However, there may be a further application of these ideas within the philosophy of science. The confirmatory practice of scientists has received a lot of attention from formally oriented philosophers of science, often with the aim of explaining or rationalizing science, or of providing scientists with norms that guide the inference from data to theory. Experimental practice, on the other hand, has not been subject to the same scrutiny by formal modelers. It has been the subject of science and technology studies, but not of formal philosophy of science. We believe formal philosophy of science will have interesting things to say about experimentation because the tools to describe interventions in mathematical terms are available. We hope that with the present study, we are contributing to the development of such a formal philosophy of experiment.

## Appendix

This appendix substantiates the claim that if the system of Eqs. (14) has a solution, then this solution is unique. We are only dealing with the specific example of this paper and do not generalize the result. The generalization will bring rather cumbersome algebraic expressions and, we believe, little added insight. The reader may glean the strategy for an analytical investigation of the solution space, and an associated proof strategy for the general case, from what follows.^14^

We first combine the expressions of Eqs. (5) and (13) to obtain

15 $$\begin{aligned} \theta _{10} + \theta _{11} &=  \delta \phi _{1} + (1 - \delta ) \phi _{0} \equiv f, \nonumber \\ \theta _{01} + \theta _{11} &=  \delta \lambda _{1} + (1 - \delta ) \lambda _{0} \equiv l, \nonumber \\ \theta _{10}' + \theta _{11}' &= \delta ' \phi _{1} + (1 - \delta ') \phi _{0} \equiv f', \nonumber \\ \theta _{01}' + \theta _{11}' &= \delta ' \lambda _{1} + (1 - \delta ') \lambda _{0} \equiv l', \end{aligned}$$

where $f = r_{10} + r_{11}$ and $l = r_{01} + r_{11}$ are the frequencies of fear and loathing observed before intervention, and $f'$ and $l'$ are those frequencies after intervention. We can now solve for $\delta$ as well as $\delta '$ by combining Eqs. (15), thus deriving the first four constraints on the parameters:

16 $$\begin{aligned} \delta &= \frac{f - \phi _{0}}{\phi _{1} - \phi _{0}} \; = \; \frac{l - \lambda _{0}}{\lambda _{1} - \lambda _{0}}, \nonumber \\ \delta ' &=  \frac{f' - \phi _{0}}{\phi _{1} - \phi _{0}} \; = \; \frac{l' - \lambda _{0}}{\lambda _{1} - \lambda _{0}}. \end{aligned}$$

The intuitive meaning is that *f* and $f'$ must both sit in between $\phi _{0}$ and $\phi _{1}$, as determined by $\delta$ and $\delta '$, and that the relative positions of *f* and $f'$ within this interval must be matched by the relative positions of *l* and $l'$ in between $\lambda _{0}$ and $\lambda _{1}$. In terms of freedom in the parameter space, there are thus two degrees of freedom left. If, for example, we fix $\phi _{0}$ and $\phi _{1}$ by hand, the values for $\lambda _{0}$ and $\lambda _{1}$ as well as the values for $\delta$ and $\delta '$ follow.

We now determine these two constraints by a further set of two equations. Consider the expressions for fear and loathing occurring together:

17 $$\begin{aligned} \theta _{11} &=  \delta \phi _{1} \lambda _{1} + (1 - \delta ) \phi _{0} \lambda _{0} \equiv c, \end{aligned}$$

18 $$\begin{aligned} \theta _{11}' &= \delta ' \phi _{1} \lambda _{1} + (1 - \delta ') \phi _{0} \lambda _{0} \equiv c'. \end{aligned}$$

We abbreviate the frequencies of them occurring together as *c* and $c'$. We can now substitute terms appearing in Eqs. (15) into Eq. (17). With some reformulation these substitutions lead to

19 $$\begin{aligned} \lambda _{0} \phi _{1} &=  f \lambda _{0} + l \phi _{1} - c, \end{aligned}$$

20 $$\begin{aligned} \lambda _{1} \phi _{0} &=  f \lambda _{1} + l \phi _{0} - c. \end{aligned}$$

We can derive the analogous expressions for the parameters by using the frequency after intervention $c'$, now substituting terms from Eqs. (15) into Eq. (18). Combining Eqs. (19) and (20) with the analogous expressions involving $c'$, we obtain

21 $$\begin{aligned} \lambda _{0} &=  \frac{l' - l}{f - f'} \phi _{1} - \frac{c' - c}{f - f'}, \end{aligned}$$

22 $$\begin{aligned} \lambda _{1} &=  \frac{l' - l}{f - f'} \phi _{0} - \frac{c' - c}{f - f'}. \end{aligned}$$

Together with the constraints of Eq. (16) these two linear relations between the $\lambda$’s and $\phi$’s are sufficient for determining all the values of the parameters.

To solve the equations we fill in the expression for $\lambda _{0}$ of Eq. (21) into Eq. (19), thereby obtaining a quadratic equation for $\phi _{1}$:

$$\begin{aligned} \left( \frac{l' - l}{f - f'} \phi _{1} - \frac{c' - c}{f - f'} \right) \phi _{1} \; = \; f \left( \frac{l' - l}{f - f'} \phi _{1} - \frac{c' - c}{f - f'} \right) + l \phi _{1} - b. \end{aligned}$$

A parallel expression for $\phi _{0}$ can be obtained by filling in $\lambda _{1}$ of Eq. (22) into Eq. (20), but if soluble within the domain [0, 1], this expression will yield the same two solutions. Once we choose either of the two solutions for $\phi _{1}$, the parameter $\phi _{0}$ takes on the other value. And once we have solved for $\phi _{1}$ and chosen whether it obtains the higher or the lower of the two values, we thereby fix the values of all the other parameters. Swapping around the two solutions will effectively swap around the ordering among $\delta$ and $\delta '$, according to the expressions above.

With respect to the interpretation of depression, fear, loathing, and treatment, the normal case will have $f' < f$, $l' < l$ and $c' < c$ so that $\lambda _{0} < \lambda _{1}$, $\phi _{0} < \phi _{1}$, and $\delta ' < \delta$. A further investigation of the space of solutions can be undertaken by identifying of each point in the space of frequencies whether or not the constraints can all be met. However, for present purposes the abstract characterization suffices, alongside the remark that the space of solutions is non-empty.

## Footnotes

1 We follow the Bayesian idea that hypotheses $H_{\theta }$ can serve as arguments of the probability function. Further conventions are that equations, like $Q_{t+1} = 1$, can appear as arguments of a probability function, and that expressions like $S_{t}$ function as variables.

2 Any infinitely long sequence of results is in principle consistent with any of the hypotheses $H_{\theta }$, and in that sense we are encountering an underdetermination problem in the estimation. However, here we will not consider this type of identifiability.

3 See Lawley and Maxwell (1971) for a classical statistical overview, Mulaik (1985) for a philosophically-minded discussion, and Bartholomew and Knott (1999) for a very insightful introduction from a Bayesian perspective. All these treatises introduce exploratory factor analysis as well as the much less problematic statistical tool of confirmatory factor analysis. In this paper we concentrate on the former, and simply call it factor analysis.

4 See Bartholomew and Knott (1999) for a general introduction. Seeing that exploratory factor analysis generates a structure that explains the observed correlations, it is rather natural that Haig (2005) and Schurz (2008) present it as a formal model of abduction.

5 This is a coordinate transformation in the space of latent variables, characterizing it in terms of different bases.

6 This criterion is known as “varimax”; see, e.g., Lawley and Maxwell (1971).

7 See Steiger (1979) for some historical context, Maraun (1996) for a philosophical evaluation, McDonald (1974) for an excellent classical statistical discussion, and Bartholomew and Knott (1999) for a Bayesian account of it.

8 There are some restrictions to this allocation. For example, as worked out in Ellis and Junker (1997), if we let the number of manifest variables increase and assume that there is a single latent variable that is tail-measurable in terms of these manifest variables, then the factor scores are determined up to a monotonic transformation.

9 See Korb et al. (2004) and Eberhardt and Scheines (2007) for further discussion of kinds of intervention.

10 We thank an anonymous reviewer for suggesting this formulation to us.

11 It is a topic of ongoing debate whether latent variables have to be taken as real in some sense (cf. Borsboom et al. 2003). This debate is relevant to our concerns, but not sufficiently to motivate an indepth discussion here. We need not adopt a realist or an instrumentalist view on latent variables to appreciate the point that theoretical criteria, formulated in terms of such latents, can be replaced by causal assumptions and intervention data. Similarly, the insightful discussion in Weinberger (2015) on latents and ideal interventions is relevant but not crucial: our points do not hinge on the interventions on latents being ideal.

12 In the statistical literature, the idea that we can confirm or disconfirm probability distributions over statistical parameters has become known as hierarchical Bayesian modelling. See, for instance, Chapter 5 of Gelman et al. (2013), and the philosophical appraisals in Henderson et al. (2010) and Romeijn (2013).

13 In many cases psychometricians will have more pressing concerns than the exact identifiability of the parameters. See, for example, Hayduk and Littvay (2012), who argue that it is often preferable to accept some uncertainty in the determination of the model. In their view it is better to use the few best indicators, and direct further observation efforts towards developing more sophisticated theoretical models, so as to bring mediating and confounding variables into view. However, this does not take away from our point that intervention data may be highly informative.
